# Reversible renal-limited thrombotic microangiopathy due to gemcitabine-dexamethasone-cisplatin therapy: a case report

**DOI:** 10.1186/s12882-021-02386-y

**Published:** 2021-05-12

**Authors:** Masashi Nishikubo, Yoshimitsu Shimomura, Nobuhiro Hiramoto, Naohiko Sawamura, Takako Yamaguchi, Shigeo Hara, Takayuki Ishikawa

**Affiliations:** 1 Department of Hematology, Kobe City Medical Center General Hospital, 2-1-1 Minami-machi, Minatojima, Chuo-ku, Kobe, Hyogo 650-0047 Japan; 2 Department of Nephrology, Kobe City Medical Center General Hospital, Kobe, Hyogo 650-0047 Japan; 3 Department of Pathology, Kobe City Medical Center General Hospital, Kobe, Hyogo 650-0047 Japan

**Keywords:** Thrombotic microangiopathies, Gemcitabine, Cisplatin, Urinalysis, Lymphoma, Proteinuria

## Abstract

**Background:**

Gemcitabine and cisplatin are chemotherapeutic agents used for treating multiple cancers, and these agents are sometimes used in combination. Drug-induced thrombotic microangiopathy (TMA) is a rare but potentially fatal complication. It typically presents as a systemic disease with the classical triad of hemolytic anemia, thrombocytopenia, and organ damage. In contrast to systemic TMA, cases of renal-limited TMA, defined as biopsy-proven renal TMA without the classical triad, have been reported with relatively good prognosis. Most cases of renal-limited TMA are associated with calcineurin inhibitors, and cases of drug-induced renal-limited TMA due to gemcitabine-dexamethasone-cisplatin therapy have been rarely reported.

**Case presentation:**

A 43-year-old woman with lymphoma developed acute kidney injury with marked proteinuria, microhematuria, and abnormal urinary casts after receiving one cycle of gemcitabine-dexamethasone-cisplatin therapy. Although she did not show hemolytic anemia and thrombocytopenia, renal biopsy showed diffuse injury to the glomerular endothelial cells, supporting the diagnosis of renal-limited TMA. Her condition improved only with the cessation of gemcitabine and cisplatin treatment. She received another chemotherapy without gemcitabine and platinum agents, and no recurrence of renal-limited TMA was observed.

**Conclusions:**

Drug-induced TMA occurs early after gemcitabine and cisplatin use in renal-limited form and is reversible when detected and managed in a timely manner. Urinalysis, which is simple and inexpensive and can be easily performed, is a beneficial screening tool for early-onset drug-induced TMA among patients who receive gemcitabine-dexamethasone-cisplatin therapy.

## Background

Drug-induced thrombotic microangiopathy (TMA) is a rare but significant complication associated with multiple drugs, including chemotherapeutic agents [1]. Gemcitabine, a deoxycytidine analog, and cisplatin, a platinum-containing agent, are widely used as chemotherapeutic agents for malignancy. The combination of gemcitabine and cisplatin is employed in some cases, such as non-small-cell lung carcinoma; hepatobiliary, pancreatic, and urothelial cancer; and relapsed or refractory non-Hodgkin lymphoma [2–7]. Both gemcitabine and cisplatin have been reported to cause drug-induced TMA [8–11]. Regarding drug-induced TMA due to gemcitabine, its incidence has been reported to range from 0.015 to 1.4% [12]. The risk increases with the dose of gemcitabine and with prolonged duration of treatment [12]. Similarly, drug-induced TMA due to cisplatin has been also reported, although it is significantly less frequent than gemcitabine [9, 13]. Drug-induced TMA is a severe complication associated with significant mortality regardless of causative agents [10, 12, 13], and renal damage possibly leading to end-stage renal disease is one of complications observed in drug-induced TMA [12]. Clinically, this condition manifests as a systemic disease, with the classical triad of hemolytic anemia, thrombocytopenia, and organ damage, including renal insufficiency [12, 13]. Apart from the systemic form of TMA, few cases of drug-induced TMA due to gemcitabine and cisplatin, localized in the kidneys, have been reported. Herein, we report a case of a 43-year-old woman with drug-induced renal-limited TMA, which developed after one cycle of gemcitabine-dexamethasone-cisplatin (GDP) therapy. Her condition improved rapidly after cessation of GDP therapy, and urinalysis was beneficial for the early detection of drug-induced TMA.

## Case presentation

**Fig. 1:**
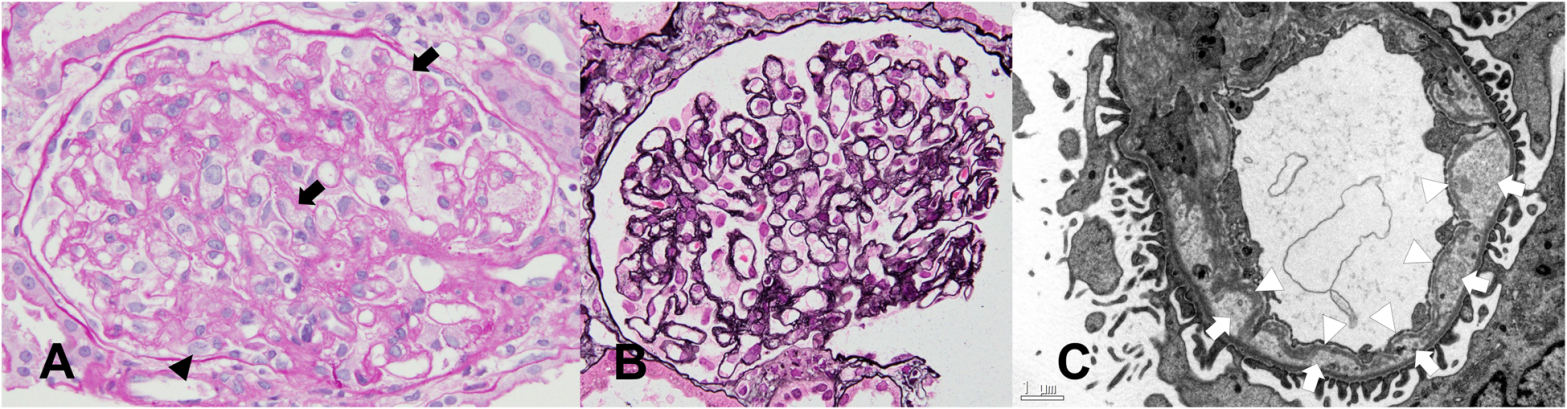
Renal biopsy finding. **a** Periodic acid-Schiff staining (× 400). The glomerulus shows diffuse endothelial swelling (a black arrowhead) and scattered foamy macrophages (black arrows). **b** Periodic acid-methenamine silver staining (×400). Reduplication of the glomerular basement membrane is observed. **c** Electron micrograph of a glomerular basement membrane (× 6000). New basement membrane formation (white arrowheads) and subendothelial edema (white arrows) are present

**Fig. 2:**
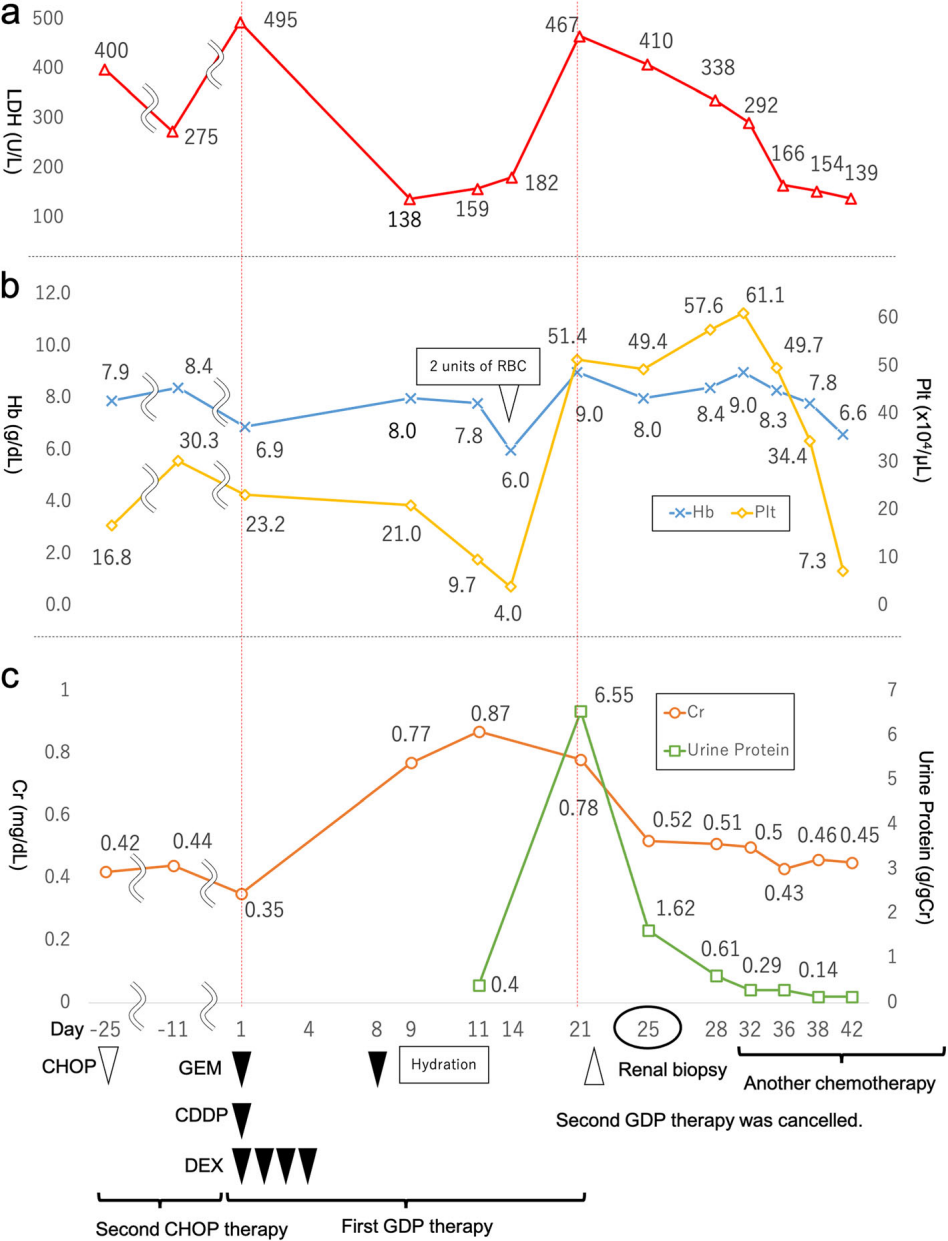
Her clinical course. Day 1 stands for the day when the first dose of gemcitabine-dexamethasone-cisplatin therapy was administered. **a** LDH levels. The elevation of LDH was observed at the beginning of each chemotherapy. It temporally resolved with chemotherapy initiation, but it worsened after the neutrophil recovery in each course. At the onset of renal-limited TMA, it was observed again probably due to lymphoma itself and inflammation with TMA. It gradually improved with the cessation of GDP therapy and got normalized after the administration of another chemotherapy. **b** Hemoglobin and platelet levels. The patient already had anemia due to chronic inflammation of refractory lymphoma and myelosuppression due to chemotherapy. However, at the onset of renal-limited TMA, her anemia did not deteriorate; rather, it improved. Also, at the onset of renal-limited TMA, thrombocytosis, not thrombocytopenia, was observed. **c** Creatinine levels, the amount of urine protein and interventions. The doses of chemotherapy were as follows: 1000 mg/m^2^ of gemcitabine on days 1 and 8, 33 mg/day of dexamethasone from day 1 to day 4, and 75 mg/m^2^ of cisplatin on day 1. Abbreviations. GEM: gemcitabine. CDDP: cisplatin. DEX: dexamethasone. RBC: red blood cells

A 43-year-old Japanese woman was admitted to our hospital for the treatment of angioimmunoblastic T-cell lymphoma. She presented with continuous high fever and a disseminated skin rash. Laboratory data showed elevated lactate dehydrogenase (LDH), C-reactive protein (CRP), and soluble-interleukin-2 receptor levels at hospitalization. She received two cycles of cyclophosphamide, vincristine, doxorubicin, and prednisolone (CHOP) therapy. Her symptoms and abnormal laboratory findings were temporally resolved with chemotherapy initiation, but these worsened after the neutrophil recovery in each course. Positron emission tomography demonstrated residual uptake of fluorodeoxyglucose in the spleen and iliac lymph nodes, suggesting the ineffectiveness of CHOP therapy. Subsequently, she received GDP therapy, which consisted of 1000 mg/m^2^ of gemcitabine on days 1 and 8, 33 mg/day of dexamethasone from day 1 to day 4, and 75 mg/m^2^ of cisplatin on day 1 [14]. She received one cycle of GDP therapy as scheduled above with 2800 mg of gemcitabine and 100 mg of cisplatin in total. She developed acute kidney injury (AKI) with a creatinine (Cr) level of 0.35 mg/dL to 0.77 mg/dL, proteinuria (402 mg/gCr), and microhematuria (1–4/high-power field of red blood cell without dysmorphic change) 9 days after the initiation of GDP therapy. Her AKI failed to improve despite hydration and worsened 21 days after the initiation of GDP therapy. Her physical examination at this time revealed a blood pressure of 118/58 mmHg, and the other vital signs were unremarkable. Laboratory findings showed anemia without signs of hemolysis and thrombocytosis and elevated liver enzyme, LDH, and CRP levels (Table 1). There were no coagulopathy and schistocytes in the peripheral blood smear. The abnormal findings of liver enzymes, LDH, and CRP were similar to the previous deteriorations observed after neutrophil recovery of each CHOP therapy and were considered to be mainly due to refractory lymphoma. Regarding the elevated LDH level, based on the absence of findings suggesting hemolysis, such as progressive anemia, elevated indirect bilirubin level, and presence of schistocytes in the peripheral blood smear, it suggested systemic inflammation, not hemolysis. Urinalysis showed marked proteinuria (6549 mg/gCr), microhematuria (30–49/high-power field of red blood cell with dysmorphic change), and hyaline, granular, waxy, and epithelial casts. A 24-h urine specimen showed a protein level of 2464 mg/day. Next, we cancelled the scheduled second GDP therapy and performed a renal biopsy. Moreover, histological findings revealed diffuse and global endothelial swelling, double contours of the glomerular basement membranes, and scattered foamy macrophages. Electron microscopic findings revealed subendothelial edema and new basement membrane formation (Fig. 1). Immune complexes were not found on immunofluorescence. Immunofluorescence staining of complement C3 and C4 was negative. Based on the history of chemotherapy and the absence of the classical triad of systemic TMA, such as hemolytic anemia, thrombocytopenia, and organ damage other than the kidneys, the patient was diagnosed with drug-induced renal-limited TMA due to GDP therapy. She was followed up carefully, and her proteinuria improved gradually. Her AKI also improved to baseline with a Cr level of 0.43 mg/dL and proteinuria of less than 500 mg/gCr 36 days after the initiation of GDP therapy. She received another chemotherapy without gemcitabine and platinating agents, and no recurrence of renal-limited TMA was observed **(**Fig. 2**)**.

**Table 1:** Laboratory findings at renal-limited thrombotic microangiopathy onset

Test	Result	Reference range
WBC (white blood cells) (/μL)	7600	3900–9800
Hemoglobin (g/dL)	9.0	11.1–15.1
PLT (platelet count) × 10^4^/μL	51.4	13.0–37.0
TP (total protein (g/dL)	5.9	6.5–8.5
Albumin (g/dL)	3.2	3.9–4.9
T-Bil (total-bilirubin) (mg/dL)	0.7	0.2–1.2
AST (aspartate aminotransferase) (U/L)	42	8–40
ALT (alanine aminotransferase) (U/L)	131	8–40
LDH (lactate dehydrogenase) (U/L)	573	124–222
Urea (mg/dL)	19.2	8.0–20.0
Creatinine (mg/dL)	0.78	0.40–0.80
CRP (C-reactive protein) (mg/dL)	10.36	0.00–0.50
PT-INR (prothrombin time-international normalized ratio)	1.10	
APTT (activated partial thromboplastin time (sec)	26.9	24.3–38.9
Fibrinogen (mg/dL)	483	180–320
Complement C3 (mg/dL)	103	65–135
Complement C4 (mg/dL)	31	13–35

## Discussion and conclusions

The clinical course of this patient raised two critical issues. First, few doses of gemcitabine and cisplatin can cause renal-limited TMA. Second, renal-limited TMA due to GDP therapy can be alleviated by treatment cessation, and urinalysis may be useful for early detection.

TMA is a pathologic disease entity characterized by generalized microvascular occlusion by platelet emboli, triggered by multiple etiologies, including medications [15]. It presents with the classical triad of thrombocytopenia, microangiopathic hemolytic anemia, and organ injury, including AKI. Apart from the systemic form of TMA, renal-limited TMA cases, defined as biopsy-proven renal TMA without the classical triad, have been reported [13]. Most renal-limited TMA cases are associated with calcineurin inhibitors, which are used for immunosuppression after kidney transplantation [16, 17]. Bevacizumab and quinine can also cause renal-limited TMA [18, 19]. In contrast, although both gemcitabine and cisplatin have been reported as causative agents of drug-induced systemic TMA, alone or in combination, few cases of renal-limited drug-induced TMA with those agents have been reported [16].

The primary side effects of GDP therapy are myelosuppression, mild liver function abnormalities, gastrointestinal symptoms, infection, thrombosis or embolism, and edema [6, 7]. Regarding gemcitabine, mild proteinuria and hematuria have been commonly reported as side effects of gemcitabine, but these are rarely clinically significant [20]. Although rare, drug-induced TMA due to gemcitabine is a severe complication [12], and the risk of systemic TMA is more significant when the cumulative dose exceeds 12,000 mg/m^2^ or when the treatment lasts longer than 7 months [12, 21]. However, lower doses of gemcitabine with or without other anticancer drugs including cisplatin can be the cause of systemic TMA [10, 22, 23]. In the present case report, renal-limited TMA developed after the patient was exposed to only 2000 mg/m^2^ of gemcitabine and 75 mg/m^2^ of cisplatin, although it is not clear to what extent each drug contributed to the development of TMA. Therefore, clinicians should consider TMA in cases with findings suggestive of TMA, including proteinuria, among patients receiving gemcitabine and cisplatin, even when the cumulative dose is not significant.

Renal-limited TMA usually presents with proteinuria or AKI, and in cases of renal transplant patients, it can be suspected with delayed graft function [16]. Minimization or temporary withdrawal of causative medications is the major therapeutic intervention, and the prognosis is relatively good, with the survival ranging from 75 to 100% [17, 24, 25]. However, once systemic drug-induced TMA associated with gemcitabine and/or cisplatin has developed, treatment cessation alone is rarely curative [12, 13]. Intensive treatment plans, such as plasma exchange, fresh frozen plasma infusion, and steroids and eculizumab administration, are indicated. Although it is still controversial, renal-limited TMA may be a prodromal stage of systemic TMA. Cases wherein TMA initially localizes to the kidney on biopsy, but subsequently progresses to manifest as systemic TMA, have been reported [26, 27]. Therefore, early recognition and prompt intervention are warranted. In the present case report, we detected renal-limited drug-induced TMA by urinalysis. Early detection prompted treatment cessation, which resulted in complete improvement of symptoms. Since urinalysis is simple, inexpensive, and easily performed, routine urinalysis may be useful for early detection and intervention with chemotherapy modification. There are some limitations in our case report. Unfortunately, we did not investigate the Coombs test, haptoglobin value, and ADAMTS-13 activity because we did not consider TMA as a differential diagnosis at the onset of TMA. We did not also perform the genetic analysis of complement genes, which is not available on a commercial basis in Japan.

In conclusion, GDP therapy caused renal-limited TMA, even when the cumulative dose of gemcitabine or cisplatin was low. With early detection and prompt cessation of causative agents, it was still reversible. Routine screening with urinalysis may be beneficial for patients receiving GDP therapy.

## Data Availability

Not applicable.

## Abbreviations

TMA: Thrombotic microangiopathy
CHOP: Cyclophosphamide, vincristine, doxorubicin, and prednisolone
GDP: Gemcitabine, dexamethasone, and cisplatin
AKI: Acute kidney injury
Cr: Creatinine
